# Human APOBEC1 cytidine deaminase edits HBV DNA

**DOI:** 10.1186/1742-4690-6-96

**Published:** 2009-10-21

**Authors:** Minerva Cervantes Gonzalez, Rodolphe Suspène, Michel Henry, Denise Guétard, Simon Wain-Hobson, Jean-Pierre Vartanian

**Affiliations:** 1 Molecular Retrovirology Unit, Virology Department, Institut Pasteur, 28 rue du Dr Roux 75724 Paris cedex 15, France

## Abstract

Retroviruses, hepadnaviruses, and some other retroelements are vulnerable to editing by single stranded DNA cytidine deaminases. Of the eleven human genes encoding such enzymes, eight have demonstrable enzymatic activity. Six of seven human APOBEC3 are able to hyperedit HBV DNA, frequently on both strands. Although human APOBEC1 (hA1) is not generally expressed in normal liver, hA1 can edit single stranded DNA in a variety of experimental assays. The possibility of ectopic expression of hA1 *in vivo *cannot be ruled out and interestingly, transgenic mice with A1 expressed under a liver specific promoter develop hepatocellular carcinoma. The impact of hA1 on HBV in tissue culture is varied with reports noting either reduced DNA synthesis or not, with cytidine deamination taking a low profile. We sought to examine the hA1 editing activity on replicating HBV. Using highly sensitive 3DPCR it was possible to show that hA1 edits the HBV minus DNA strand as efficiently as hA3G, considered the reference deaminase for HIV and HBV. The dinucleotide specificity of editing was unique among human cytidine deaminases providing a hallmark of use in *a posteriori *analyses of *in vivo *edited genomes. Analysis of sequences derived from the serum of two chronic carriers, indicated that hA1 explained only a small fraction of edited HBV genomes. By contrast, several human APOBEC3 deaminases were active including hA3G.

## Findings

Despite being the prototypic human cytidine deaminase, human APOBEC1 (hA1) first identified in 1995 has been overshadowed by other paralogs, notably activation induced cytidine deaminase (AICDA) and the APOBEC3 gene cluster at ch22q13.1 [1-3]. Human A1 (hA1) edits a single cytidine residue in human apolipoprotein B (apoB) mRNA, a specificity that is conferred by its major interactor, ACF, its expression being confined to intestinal epithelial cells [4,5]. By contrast, the mouse, rat, dog and horse A1s are expressed in the intestine and other organs including the liver [2,6,7]. This situation is probably due to an Alu insertion in a part of the hA1 gene inactivating a generalist promoter [8,9]. RNA editing specificity can break down in rabbit A1 transgenic mice where hyperediting of the apoB mRNA was described [10] and subsequently noted in normal mouse intestinal tissues [11]. For transgenic mice expressing the rabbit APOBEC1 gene under the control of a liver specific promoter, hepatic dysplasia and hepatocellular carcinomas were found [12]. Whether this is due to RNA or DNA editing is unknown although in an *E. coli *DNA mutator assay, hA1 was highly mutagenic meaning that the latter cannot be ruled out [13]. It turned out that human and mouse/rat A1 enzymes are not true orthologs in that the rodent enzymes can hyperedit both RNA and DNA, unlike hA1 that can only deaminate ssDNA [13]. By contrast AICDA and the human APOBEC3 (hA3) show an exclusive single stranded DNA substrate specificity [14-27].

As retroviruses and hepadnaviruses replicate via a single stranded cDNA intermediate, it is not surprising that some are vulnerable to the effects of these cytidine deaminases if expressed in the target cell. While there is a huge literature on the interaction between human immunodeficiency virus (HIV) and the hA3 cluster of genes (the hA3 genes), most of them can also edit hepatitis B virus (HBV) DNA [28-33]. The role of A1 genes on retroviral replication is somewhat checkered. One report has shown that HBV replication is restricted by hA1 yet doesn't address the question of editing [34]. By contrast, another study shows that the rat A1 deaminase hardly impacts HBV replication, even though a little cytidine deamination was found [30]. Both hA1 and rat A1 impact HIV replication in single cycle growth assays [35]. In the mouse A1 hardly restricts Friend murine leukemia virus replication, although using highly sensitive 3DPCR hypermutants were recovered from a small fraction of cultured cells or splenocytes from infected mice [11].

**Figure 1:**
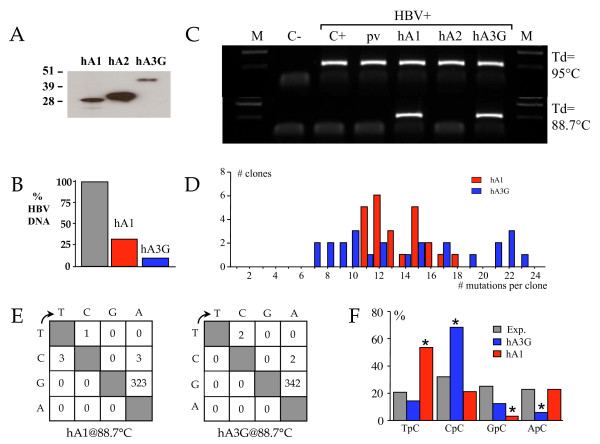
**Human APOBEC1 can efficiently hyperedit HBV minus strand DNA**. A) Western blotting of V5 tagged hA1, hA2 and hA3G cDNA constructs. Molecular weight markers (kDa) are given to the left. B) Reduction of HBV DNA production following cotransfection of QT6 cells with HBV ± hA1 ± hA3G. C) PCR and 3DPCR of HBV X region DNA from transfections. C-, no DNA control; C+, HBV alone; pv, plasmid vector control; hA1, V5-tagged human APOBEC1 expression plasmid; hA2, V5 tagged human APOBEC2; hA3G, V5-tagged human APOBEC3G as positive control. M, molecular weight markers. The sizes of the PCR and 3DPCR fragments are 314 and 213 bps. D) Frequency distribution of G->A hypermutants in terms of mutations per clone. E) Mutation matrices of 24 hA1 and hA3G hyperedited HBV sequences. The size of the locus is 167 bp. F) Dinucleotide analysis of hA1 and hA3G edited HBV genomes. A χ^2 ^analysis showed that frequencies for hA1 and hA3G deviated significantly from the expected values as indicated by asterisks (p < 0.001).

We sought to investigate the editing capacity of hA1 on HBV replication using 3DPCR [28,31,32]. An infectious molecular clone of HBV was transfected into quail QT6 cells with hA1 along with human APOBEC2 (hA2) and human APOBEC3G (hA3G) as negative and positive controls. QT6 cells were used so as to eliminate the endogenous APOBEC background typical of mammalian and human cell lines [28]. At 72 h culture supernatants and cell lysates were recovered. Western blotting of whole cell lysates showed that all three APOBEC constructs were produced without obvious degradation (Figure 1A), the levels of hA1 and hA3G being particularly comparable. Using SYBR green quantification of HBV DNA, when normalized to the empty expression vector transfection control, hA1 and hA3G clearly impacted DNA replication (Figure 1B). PCR was performed on the HBV X gene using a conventional two-step procedure as previously described [28,31]. Amplification performed using a 95°C denaturation temperature recovered HBV DNA in all samples, apart from the negative DNA control (Figure 1C). When 3DPCR was performed on these PCR products at the restricting temperature of 88.7°C, DNA was recovered only from the hA1 and hA3G transfections (Figure 1C). Cloning and sequencing of 24 clones identified so-called G->A hypermutants indicative of genetic editing of the minus DNA strand or cDNA, the frequency distribution of edited genomes per sequence being generally comparable (Figure 1D). No C->T hypermutants, indicative of plus strand editing, were recovered. The mutation matrices (Figure 1E) and editing frequencies were strictly comparable, hA1 32% (range 16-52%) while that for hA3G was 34% (range 25-41%). In short, hA1 is every bit as efficient as hA3G in editing HBV DNA.

As the dinucleotide context of editing is frequently a hallmark of different deaminases, for example hA3G shows a strong preference for CpC, while hA3H prefers TpC, the hA1 edited genomes were so analyzed. As can be seen from Figure 1F, hA1 showed a strong preference for TpC and a strong aversion for GpC. It was neutral with respect to ApC. All other hA3 deaminases (hA3s) either avoid GpC and ApC or are neutral (hA3A, hA3C, [28]). Hence, along with the preference for TpC, this feature is highly distinctive of hA1 editing.

**Figure 2:**
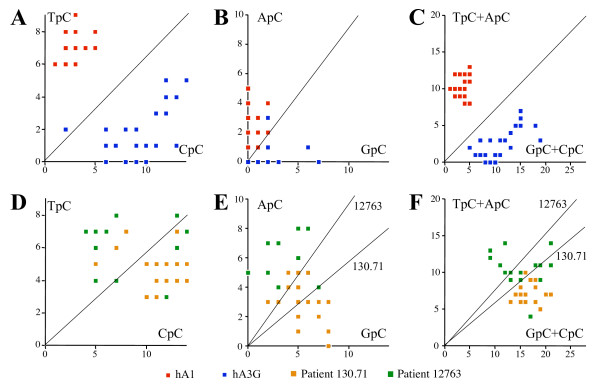
**Single molecule analysis of hyperedited HBV DNA**. Based on the macroscopic trends identified in Figure 1F, mutated dinucleotide frequencies were plotted for individual minus strand hyperedited sequences. The units are numbers of NpC edited sites per clone. A-C) Data from HBV co-transfections with hA1 and hA3G (24 sequences each). D-F) Patient data from two individuals with high viremia chronic hepatitis (#12763, 19 sequences, ~10^9 ^DNA copies/mL; #130.71, 21 sequences, ~3 10^9 ^DNA copies/mL). For A to F, a few data points overlap; hence the number of points is less than the number of sequences. The diagonals represent the expected values assuming no dinucleotide preference. As the base composition differs between the sequences of the two HBV strains, dinucleotide composition varies and the expected values accordingly.

Of course *in vivo*, one or more APOBEC deaminases could be operative, either in different HBV infected cells or even within the same cell. Hence global dinucleotide analyses of edited genomes from patients might be misleading. Comparative analysis of the dinucleotide contexts for individual *in vivo *hyperedited sequences with reference sets derived from transfections with individual hA1 or hA3 genes should help highlight those deaminases operative *in vivo*. The only exception would be if two different hA3 enzymes were packaged in the same virion. As can be seen plotting the number of edited TpC vs. CpC sites allows a clear distinction between hA1 and hA3G edited sequences (Figure 2A). The same is true for GpC vs. ApC (Figure 2B), although the number of deaminated bases is far fewer, meaning that the observation is a little less robust. The distinction between hA1 and hA3G could be emphasized by analyzing TpC+ApC (unique to hA1) vs. GpC+CpC (Figure 2C).

We have previously reported hA3 editing of HBV genomes from sera derived from two chronically infected patients with high viremia (> 10^9 ^DNA copies/ml, [31]). Single molecule analysis of these sequences showed that there was more overlap between the patient data "clouds" than with that of hA3G. Although the individual dinucleotide analyses might suggest that a few genomes are edited by hA1 *in vivo *(Figures 2D, E vs. 2A, B), the combined dinucleotide analysis (Figure 2C vs. 2F) showed no overlap with the hA1 edited sequence set. We have previously shown that the dinucleotide bias for individual hA3 deaminases varies with the base composition of the locus [28]; hence, it is not possible to extend the analysis to other edited regions of the HBV genome.

As ~20 hypermutated sequences were derived from each of the sera, the resolution of the argument is ~5%. Obviously with 10 times more patient sequences it might be able to find *bona fide *evidence of a little hA1 editing. By contrast, hA3G accounts for a sizeable fraction (79% and 58% respectively for patients 130.71 and 12763). Accordingly, other hA3 deaminases must be involved; yet given the similarities between the editing contexts of hA3A, hA3B and hA3C, it is not possible to be more precise. Furthermore, chronic hepatitis shows many facets; and although hA1 expression is confined to the intestinal epithelium and not the liver [6], its expression profile in a variety of different clinical presentations including highly inflamed cirrhotic tissue is not known.

As mentioned above, hA1 transgenic mice under the control of the liver specific apoE promoter presented with hepatic dysplasia and ultimately liver cancer; thus, the role of cytidine deamination remains open. Even though hA1 does not edit HBV at a high frequency, because hA1 can shuttle between the cytoplasm and nucleus, it is potentially a more likely pro-cancerous candidate than hA3G, which is strictly cytoplasmic.

## Abbreviations

3DPCR: Differential DNA denaturation PCR; ACF: APOBEC1 complementary factor; hAICDA: Human activation induced cytidine deaminase; hAPOBEC1, hA1: Human apolipoprotein B editing complex protein 1; hAPOBEC2, hA2: Human apolipoprotein B editing complex protein 2; hAPOBEC3, hA3: Human apolipoprotein B editing complex protein 3; hAPOBEC3A-H, hA3A-H: Human apolipoprotein B editing complex protein 3 proteins A to H; HBV: Hepatitis B virus; HIV-1: Human Immunodeficiency virus type 1.

## Competing interests

The authors declare that they have no competing interests.

## Authors' contributions

MCG, MH, DG and RS performed the work. SWH and JPV designed the study and wrote the paper.
